# Monkeypox and measles: two scourges among children displaced by wars in the eastern region of the Democratic Republic of Congo

**DOI:** 10.1097/MS9.0000000000003897

**Published:** 2025-09-15

**Authors:** Christian Tague, Amidu Alhassan, Maher Ali Rusho, Areeba Aamir Ali Basaria, Ismat Fatima, Hermann Yokolo, Joshua Ekouo, Dujardin Makeda, Fabien Imani Shangalume, Hugues Cakirwa, Samson Hangi, Elie Kihanduka, Jones Onesime, Excellent Rugendabanga, Aymar Akilimali, Criss Koba Mjumbe

**Affiliations:** a Department of research, Medical Research Circle (MedReC), Goma, DR Congo; b College of Health and Allied Sciences, School of Nursing and Midwifery, University of Cape Coast, Cape Coast, Ghana; c Department of medical biophysics, University of Toronto, Toronto, Canada; d Department of Medicine, Dow University of Health Sciences, Karachi, Pakistan; e Department of Medicine, Jinnah Sindh Medical University, Karachi, Pakistan; f Faculty of Medicine, University of Lubumbashi, Lubumbashi, DR Congo

Monkeypox (Mpox) and measles are viral skin infections caused by *orthopoxviral and morbillivirus*, respectively; they are two of the most infectious viruses spread through contact with an infected person. Some of the signs and symptoms of both diseases include rash, cough, fever, and exhaustion^[1]^. The two diseases, Mpox, and measles epidemics share a common characteristic that they occur in populations with precarious living conditions such as refugees living in camps. These two are real public health problems of the developing countries of sub-Saharan Africa, and the Democratic Republic of Congo (DR Congo). Following the armed conflicts in Eastern Congo, the local population has been highly internally displaced into the refugee camps for the last two decades, further creating an entourage of promiscuity and congestion within the displaced centers or camps, hence decreased hygiene practices due to a small amount of access to drinking water and quality sanitation facilities. All of these, besides access to vaccination, make children susceptible to infectious diseases such as measles and Mpox^[2]^.

For decades, DR Congo has experienced several epidemics of measles across the country’s territory and more specifically in refugee camps. Indeed, these camps represent a welcome context for the spread of the disease of the precarious living conditions implied by these populations, more specifically children who are most vulnerable. In 2024, the country reported a total of 9400 cases of Mpox nearly with 399 deaths for an estimated mortality rate of 4.2%^[3,4]^.

Between 2018 and 2020, there were about 458 156 suspected cases of measles that had been registered in the DR Congo, with about 8127 deaths, representing 1.77%^[5]^. Children were the most affected; the peak of cases was recorded in 2019. Many socio-economic and environmental factors favor the propagation of epidemic diseases within sub-Saharan African countries. The Mpox and measles epidemics find a high means of propagation inside displaced persons camps. Frequent migration to camps and between various camps; the status of an overpopulated area in continuous growth; promiscuity is high, and very often this level of promiscuity is joined by precarious hygiene conditions, which, in our century, is considered one of the measures for quick saturation of infectious diseases from one area to another^[6]^.

According to some studies, the smallpox vaccine provides protection against Mpox with an overall favorable safety profile and a low incidence of adverse events. However, the reported efficacy varies considerably, ranging from 35% to 85%, depending on the populations studied, the type of vaccine used, the vaccination schedule, and specific epidemiological settings. Based on these results, several countries have authorized the use of the smallpox vaccine as an emergency measure to prevent the transmission of Mpox, particularly in high-risk groups. However, it should be noted that the variability in the data highlights the need for additional studies to better evaluate the efficacy and safety of the vaccine in different settings^[1]^.

Mpox transmission patterns vary greatly between urban and refugee environments. While sexual contact or travel-related infections may be a factor in urban transmission, skin contact and sharing bedding among youngsters are the main ways from which the disease spreads in camps. Understanding this distinction is vital for targeted interventions^[7]^.

On the other hand, living in a state of poverty cannot provide quality care for these children and their parents, hence leading to a vaccination deficit in these children. Also, frequent displacement complicates vaccination campaigns. The same low socio-economic state induces the consumption and handling of wild meat, especially primates, which are natural carriers of the virus responsible for Mpox disease. Related to this, the ignorance and health education amongst the displaced people communities lack knowledge on preventive measures and clinical signs of measles and Mpox^[8]^.

Regarding such outbreaks of Mpox and measles, which also cause devastation of living conditions among the overall displaced populations, especially children, in eastern DR Congo, it is very relevant to make vaccination against measles and Mpox widely available to such children through adequate quantities of vaccines^[4,5]^. Hence, children will require to be educated, but mainly their parents will need education about the consequences and dangers these diseases represent for their children, insisting especially on modes of transmission, the means of prevention of measles and Mpox, the importance of vaccination, and the vital necessity of declaring new cases for proper management to avoid the spread of these diseases to other children^[8,9]^. This must be done by improving the living conditions of the populations and by providing them with appropriate access to drinking water and sanitary facilities of quality to improve hygiene and cleanliness conditions. In addition to epidemiological measures to prevent and control these diseases, it is also essential to raise awareness among parents and children about the potential psychosocial impacts. Refugee children with these diseases may be exposed to stigma and discrimination, which can affect their mental health. Appropriate education can help reduce these negative effects by promoting understanding, inclusion, and psychosocial support within communities^[2,9]^.

These encounters can result in trauma, anxiety, and sadness, which are exacerbated by stigma. Programs for social reintegration and mental health counseling should be part of support networks. This is consistent with the idea of “vital metamorphosis,” in which a child’s self-identity and social role are redefined due to health changes.

In conclusion, addressing the measles and Mpox epidemics among displaced children in DR Congo requires a multidimensional approach involving expanded vaccine access, tailored health education, integration of mental health services, and infrastructural improvements. Strategic collaborations between local governments, NGOs, and international bodies are vital to implement sustainable, culturally appropriate, and effective health interventions.

## Data Availability

Not applicable.

## References

[1] Liu H Wang W Zhang Y. Global perspectives on smallpox vaccine against monkeypox: a comprehensive meta-analysis and systematic review of effectiveness, protection, safety and cross-immunogenicity. Emerg Microbes Infect 2024;13:2387442. 39082272 10.1080/22221751.2024.2387442 PMC11332295

[2] World Health Organization. Mpox. Who.int. 2024. Accessed 20 April 2025. https://www.who.int/news-room/questions-and-answers/item/mpox

[3] Agha RA Mathew G Rashid R. Transparency In The reporting of Artificial INtelligence – the TITAN guideline. Prem J Sci. 2025;10:100082.

[4] Boisson-Walsh A. Escalating Mpox epidemic in DR Congo. Lancet Infect Dis. 2024;24:e487. 38996570 10.1016/S1473-3099(24)00446-8

[5] Sodjinou VD Mengouo MN Douba A. Epidemiological characteristics of a protracted and widespread measles outbreak in the Democratic Republic of Congo, 2018-2020. Pan Afr Med J 2022;42:282. 36405650 10.11604/pamj.2022.42.282.34410 PMC9636736

[6] Jamil H Idrees M Idrees K. Socio-demographic determinants of monkeypox virus preventive behavior: a cross-sectional study in Pakistan. PLoS One 2023;18:e0279952. 37561764 10.1371/journal.pone.0279952 PMC10414588

[7] Andrasik MP Maunakea AK Oseso L. Awakening: the unveiling of historically unaddressed social inequities during the COVID-19 pandemic in the United States. Infect Dis Clin North Am 2022;36:295–308. 35636901 10.1016/j.idc.2022.01.009 PMC8806123

[8] Chaix E Boni M Guillier L. Risk of monkeypox virus (MPXV) transmission through the handling and consumption of food. Microb Risk Anal 2022;22:100237. 36320929 10.1016/j.mran.2022.100237 PMC9595349

[9] Manus JM. Retour de la rougeole, 40 millions d’enfants vulnérables. Rev Francoph Des Lab. 2023;2023:12. 10.1016/S1773-035X(23)00011-4 PMC9888019 36743943

[10] Soheili M Nasseri S Afraie M. Monkeypox: virology, pathophysiology, clinical characteristics, epidemiology, vaccines, diagnosis, and treatments. J Pharm Pharm Sci 2022;25:297–322. 36130588 10.18433/jpps33138

